# 3-Benzhydryl-1,3,4-thia­diazole-2(3*H*)-thione

**DOI:** 10.1107/S1600536813021867

**Published:** 2013-08-10

**Authors:** Megan T. Thornton, Peter C. Healy, Luke C. Henderson

**Affiliations:** a Strategic Research Centre for Biotechnology, Chemistry and Systems Biology, Deakin University, Vic 3216, Australia; b Queensland Micro and Nanotechnology Centre, Griffith University, Brisbane 4111, Australia; c School of Chemistry, Physics & Mechanical Engineering, Queensland University of Technology, Brisbane 4001, Australia

## Abstract

In the title compound, C_15_H_12_N_2_S_2_, the two phenyl rings and the planar (r.m.s. deviation = 0.002 Å) thia­diazole ring adopt a propeller conformation about the central C—H axis with H—C—C—C(phen­yl) torsion angles of 44 and 42° and an H—C—N—C(thia­diazole) torsion angle of 28°. Intra­molecular C—H⋯S and C—H⋯N contacts are observed. In the crystal, centrosymmetrically related mol­ecules associate through C—H⋯π inter­actions. These are connected into a supra­molecular chain along [101] by C—H⋯N inter­actions.

## Related literature

For details of the use of 1,3,4-thia­diazo­les in the synthesis of crown ethers, see: Pappalardo *et al.* (1987 ▶). For their uses as scaffolds in potential pharmaceuticals, see; Aggarwal *et al.* (2012 ▶); Bhole & Bhusari (2011 ▶); Ghani & Ullah (2010 ▶); Kadi *et al.* (2010 ▶); Zhan *et al.* (2009 ▶).
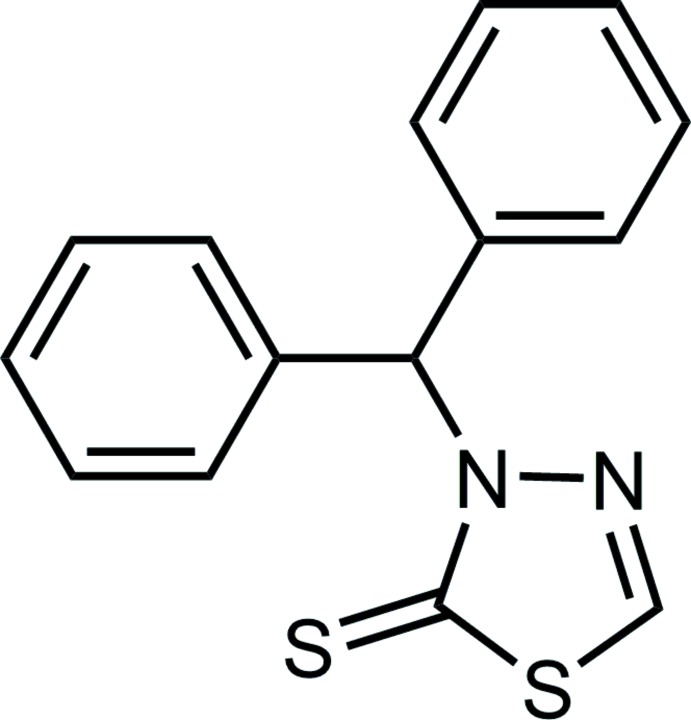


## Experimental

### 

#### Crystal data

C_15_H_12_N_2_S_2_
*M* *_r_* = 284.41
Monoclinic, 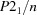

*a* = 9.1198 (4) Å
*b* = 15.4226 (5) Å
*c* = 10.7584 (4) Å
β = 108.546 (5)°
*V* = 1434.60 (10) Å^3^
*Z* = 4
Mo *K*α radiation
μ = 0.36 mm^−1^
*T* = 223 K
0.44 × 0.29 × 0.18 mm

#### Data collection

Oxford-Diffraction GEMINI S Ultra diffractometer
Absorption correction: multi-scan (*CrysAlis PRO*; Agilent, 2012 ▶) *T*_min_ = 0.859, *T*_max_ = 0.938
5244 measured reflections
2524 independent reflections
2144 reflections with *I* > 2σ(*I*)
*R*_int_ = 0.023

#### Refinement

*R*[*F* ^2^ > 2σ(*F* ^2^)] = 0.043
*wR*(*F* ^2^) = 0.108
*S* = 1.13
2524 reflections
172 parameters
H-atom parameters constrained
Δρ_max_ = 0.24 e Å^−3^
Δρ_min_ = −0.53 e Å^−3^

### 

Data collection: *CrysAlis PRO* (Agilent, 2012 ▶); cell refinement: *CrysAlis PRO*; data reduction: *CrysAlis PRO*; program(s) used to solve structure: *TEXSAN* (Molecular Structure Corporation, 2001 ▶) and *SIR97* (Altomare *et al.*, 1999 ▶); program(s) used to refine structure: *TEXSAN* and *SHELXL97* (Sheldrick, 2008 ▶); molecular graphics: *ORTEP-3 for Windows* (Farrugia, 2012 ▶); software used to prepare material for publication: *PLATON* (Spek, 2009 ▶).

## Supplementary Material

Crystal structure: contains datablock(s) global, I. DOI: 10.1107/S1600536813021867/tk5245sup1.cif

Structure factors: contains datablock(s) I. DOI: 10.1107/S1600536813021867/tk5245Isup2.hkl

Supplementary material file. DOI: 10.1107/S1600536813021867/tk5245Isup3.cml

Additional supplementary materials:  crystallographic information; 3D view; checkCIF report

## Figures and Tables

**Table 1:** Hydrogen-bond geometry (Å, °) *Cg*2 is the centroid of the C21–C26 phenyl ring.

*D*—H⋯*A*	*D*—H	H⋯*A*	*D*⋯*A*	*D*—H⋯*A*
C1—H1⋯S2	0.95	2.76	3.181 (2)	108
C16—H16⋯N3	0.95	2.58	2.897 (3)	100
C5—H5⋯*Cg*2^i^	0.95	2.74	3.670 (3)	157
C13—H13⋯N4^ii^	0.95	2.60	3.495 (3)	157

Symmetry codes: (i) 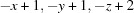; (ii) 
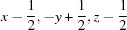
.

**Fig. 1.**
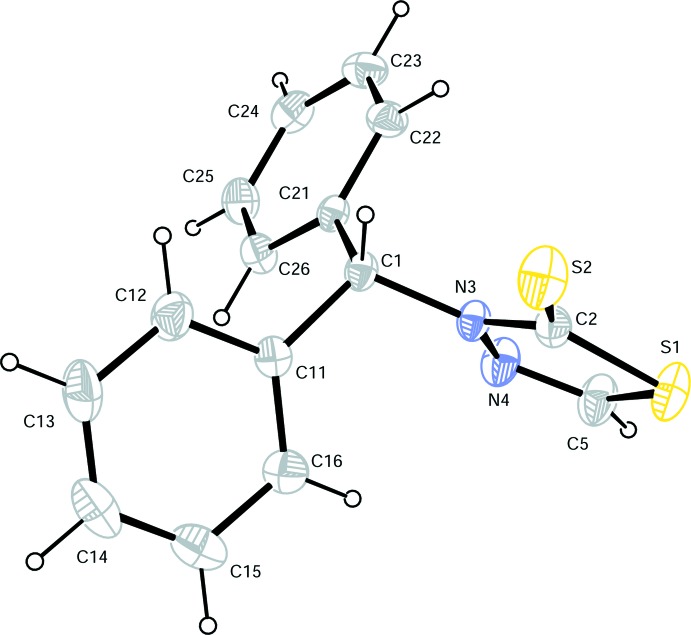
The molecular structure of the title compound with atom labelling and displacement ellipsoids for non-H atoms drawn at the 40% probability level. Hydrogen atoms are shown as spheres of arbitrary radius.

**Fig. 2.**
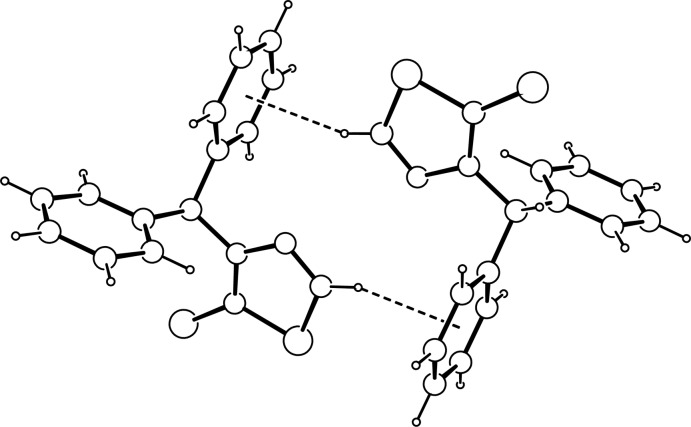
Centrosymmetrically related molecules of (I) through C—H···π inter-molecular interactions between C5—H5 and phenyl ring 2.

## supplementary crystallographic information

### 1. Comment

The structure of the title compound, (I), was determined as part of an ongoing project developing diphenylmethyl (DPM) thioethers which may be used to access sulfur-based heterocycles or as a protecting group in organic chemistry. This compound was the result of a side reaction in the synthesis of DPM thioethers, whereby the 1,3,4-thiadiazole compound is formed. The 1,3,4-thiadiazole core of the title compound is a central component of key compounds which have been used in the synthesis of crown ethers (Pappalardo *et al.*, 1987), as well as included in potential pharmacological compounds, including anti-microbials (Aggarwal *et al.*, 2012), anti-tumor compounds (Bhole & Bhusari, 2011), tyrosinase inhibitors (Ghani & Ullah, 2010), anti-inflammatory compounds (Kadi *et al.*, 2010), and HIV-1 reverse transcriptase inhibitors (Zhan *et al.*, 2009).

In (I) the two phenyl rings and the planar thiadiazole ring adopt a propeller conformation about the central C—H axis with the torsion angles H1—C1—C2—C3 = 42°, H1—C1—C8—C9 = 44° and H1—C1—N1—C2 = 28° (Fig. 1). Intra-molecular C—H···S and and both intra- and inter molecular C—H···N contacts are observed (Table 1). In the crystal lattice, centrosymmetrically related molecules associate through C—H···π inter-molecular interactions between C5—H5 and phenyl ring 2 (Table 1, Fig. 2).

### 2. Experimental

Diphenylmethanol (100 mg) was placed into a microwave reactor vessel charged with 2-mercapto-1,3,4-thiadiazole (0.1 ml) and stirred with acid-doped triethylamine:methanesulfonic acid (TeaMs, 0.25 ml). The vessel was then heated to 100°C for 20 minutes. The solution was then diluted with water and diethyl ether, 5 ml of NaOH (2*M* solution) was added and the aqueous phase extracted 3 times with diethyl ether. The combined organic phases were then dried (MgSO_4_), filtered and the solvent removed *in vacuo* to give a clear oil. Crystals of (I) suitable for single-crystal X-ray analysis were grown by slow evaporation of a solution in toluene. ^1^H NMR (400 MHz, CDCl_3_): *δ* = 8.24 (1*H*, s, thiadiazole CH), 7.71 (1*H*, s, C*H*Ph_2_), 7.42–7.22 (10*H*, m, 2 × Ph). ^13^C NMR (100 MHz, CDCl_3_): *δ* = 185.8, 143.7, 143.5, 137.8, 129.0–128.0 (10 × C), 65.8, 65.6. M.Pt: 402.2–403.2 K. HRMS, *m/z* calcd for (C_15_H_13_N_2_S_2_) 285.0514, found 285.0532.

### 3. Refinement

The carbon-bound H atoms were constrained as riding atoms with C—H = 0.95 Å. *U*_iso_(H) values were set at 1.2*U*_eq_ of the parent C atom.

### Crystal data

**Table:**

C_15_H_12_N_2_S_2_	*F*(000) = 592
*M**_r_* = 284.41	*D*_x_ = 1.317 Mg m^−^^3^
Monoclinic, *P*2_1_/*n*	Mo *K*α radiation, λ = 0.71070 Å
Hall symbol: -P 2yn	Cell parameters from 2095 reflections
*a* = 9.1198 (4) Å	θ = 3.3–30.4°
*b* = 15.4226 (5) Å	µ = 0.36 mm^−^^1^
*c* = 10.7584 (4) Å	*T* = 223 K
β = 108.546 (5)°	Block, colourless
*V* = 1434.60 (10) Å^3^	0.44 × 0.29 × 0.18 mm
*Z* = 4	

### Data collection

**Table:**

Oxford-Diffraction GEMINI S Ultra diffractometer	2524 independent reflections
Radiation source: Enhance (Mo) X-ray Source	2144 reflections with *I* > 2σ(*I*)
Graphite monochromator	*R*_int_ = 0.023
Detector resolution: 16.0774 pixels mm^-1^	θ_max_ = 25.0°, θ_min_ = 3.3°
ω and φ scans	*h* = −9→10
Absorption correction: multi-scan (*CrysAlis PRO*; Agilent, 2012)	*k* = −15→18
*T*_min_ = 0.859, *T*_max_ = 0.938	*l* = −12→8
5244 measured reflections	

### Refinement

**Table:**

Refinement on *F*^2^	Primary atom site location: structure-invariant direct methods
Least-squares matrix: full	Secondary atom site location: difference Fourier map
*R*[*F*^2^ > 2σ(*F*^2^)] = 0.043	Hydrogen site location: inferred from neighbouring sites
*wR*(*F*^2^) = 0.108	H-atom parameters constrained
*S* = 1.13	*w* = 1/[σ^2^(*F*_o_^2^) + (0.0518*P*)^2^ + 0.4906*P*] where *P* = (*F*_o_^2^ + 2*F*_c_^2^)/3
2524 reflections	(Δ/σ)_max_ = 0.001
172 parameters	Δρ_max_ = 0.24 e Å^−^^3^
0 restraints	Δρ_min_ = −0.53 e Å^−^^3^

### Special details

**Table:**

Geometry. Bond distances, angles *etc*. have been calculated using the rounded fractional coordinates. All su's are estimated from the variances of the (full) variance-covariance matrix. The cell e.s.d.'s are taken into account in the estimation of distances, angles and torsion angles
Refinement. Refinement of *F*^2^ against ALL reflections. The weighted *R*-factor *wR* and goodness of fit *S* are based on *F*^2^, conventional *R*-factors *R* are based on *F*, with *F* set to zero for negative *F*^2^. The threshold expression of *F*^2^ > σ(*F*^2^) is used only for calculating *R*-factors(gt) *etc*. and is not relevant to the choice of reflections for refinement. *R*-factors based on *F*^2^ are statistically about twice as large as those based on *F*, and *R*- factors based on ALL data will be even larger.

### Fractional atomic coordinates and isotropic or equivalent isotropic displacement parameters (Å^2^)

**Table:**

	*x*	*y*	*z*	*U*_iso_*/*U*_eq_	
S1	0.14225 (8)	0.60316 (4)	0.93721 (6)	0.0449 (2)	
S2	−0.07408 (7)	0.55846 (4)	0.66718 (7)	0.0443 (2)	
N3	0.18984 (19)	0.47793 (10)	0.80719 (16)	0.0252 (5)	
N4	0.3130 (2)	0.47539 (12)	0.92075 (18)	0.0356 (6)	
C1	0.1813 (2)	0.41149 (13)	0.70617 (19)	0.0243 (6)	
C2	0.0829 (2)	0.54127 (13)	0.7940 (2)	0.0286 (7)	
C5	0.3017 (3)	0.53775 (16)	0.9967 (2)	0.0413 (8)	
C11	0.0918 (2)	0.33159 (13)	0.7227 (2)	0.0258 (6)	
C12	0.0312 (3)	0.27892 (15)	0.6142 (2)	0.0389 (8)	
C13	−0.0465 (3)	0.20345 (16)	0.6232 (3)	0.0495 (9)	
C14	−0.0660 (3)	0.18047 (15)	0.7410 (3)	0.0500 (9)	
C15	−0.0069 (3)	0.23250 (17)	0.8493 (3)	0.0463 (9)	
C16	0.0723 (3)	0.30800 (15)	0.8406 (2)	0.0358 (7)	
C21	0.3436 (2)	0.39221 (12)	0.70245 (19)	0.0238 (6)	
C22	0.4181 (3)	0.45525 (14)	0.6523 (2)	0.0312 (7)	
C23	0.5672 (3)	0.44091 (15)	0.6484 (2)	0.0361 (7)	
C24	0.6424 (3)	0.36380 (16)	0.6942 (2)	0.0365 (8)	
C25	0.5692 (3)	0.30087 (14)	0.7442 (2)	0.0363 (7)	
C26	0.4200 (2)	0.31507 (13)	0.7485 (2)	0.0307 (7)	
H1	0.12570	0.43650	0.62400	0.0290*	
H5	0.37650	0.54700	1.08020	0.0500*	
H12	0.04310	0.29490	0.53260	0.0470*	
H13	−0.08640	0.16740	0.54840	0.0590*	
H14	−0.12000	0.12890	0.74730	0.0600*	
H15	−0.02040	0.21670	0.93030	0.0560*	
H16	0.11320	0.34360	0.91580	0.0430*	
H22	0.36700	0.50830	0.62050	0.0370*	
H23	0.61760	0.48430	0.61410	0.0430*	
H24	0.74410	0.35420	0.69130	0.0440*	
H25	0.62060	0.24780	0.77560	0.0440*	
H26	0.37010	0.27160	0.78330	0.0370*	

### Atomic displacement parameters (Å^2^)

**Table:**

	*U*^11^	*U*^22^	*U*^33^	*U*^12^	*U*^13^	*U*^23^
S1	0.0499 (4)	0.0387 (4)	0.0422 (4)	0.0092 (3)	0.0090 (3)	−0.0159 (3)
S2	0.0313 (3)	0.0448 (4)	0.0473 (4)	0.0123 (3)	−0.0008 (3)	−0.0057 (3)
N3	0.0253 (9)	0.0241 (9)	0.0232 (9)	0.0009 (7)	0.0035 (7)	−0.0041 (7)
N4	0.0382 (11)	0.0352 (10)	0.0241 (10)	0.0063 (8)	−0.0033 (8)	−0.0048 (8)
C1	0.0250 (10)	0.0234 (10)	0.0218 (10)	0.0005 (8)	0.0036 (8)	−0.0037 (8)
C2	0.0291 (11)	0.0247 (11)	0.0327 (12)	0.0003 (8)	0.0109 (10)	−0.0023 (9)
C5	0.0474 (15)	0.0391 (13)	0.0294 (13)	0.0047 (11)	0.0009 (11)	−0.0089 (10)
C11	0.0200 (10)	0.0256 (11)	0.0305 (11)	0.0015 (8)	0.0063 (9)	−0.0012 (8)
C12	0.0386 (13)	0.0395 (13)	0.0384 (13)	−0.0091 (11)	0.0119 (11)	−0.0108 (11)
C13	0.0440 (15)	0.0361 (14)	0.0647 (18)	−0.0127 (11)	0.0120 (13)	−0.0172 (13)
C14	0.0388 (14)	0.0298 (13)	0.081 (2)	−0.0030 (11)	0.0184 (14)	0.0075 (13)
C15	0.0426 (15)	0.0446 (15)	0.0529 (16)	0.0011 (11)	0.0171 (13)	0.0166 (12)
C16	0.0327 (12)	0.0386 (13)	0.0352 (13)	−0.0014 (10)	0.0094 (10)	0.0007 (10)
C21	0.0246 (10)	0.0249 (10)	0.0197 (10)	−0.0011 (8)	0.0040 (8)	−0.0044 (8)
C22	0.0337 (12)	0.0317 (12)	0.0267 (12)	0.0004 (9)	0.0076 (10)	0.0053 (9)
C23	0.0338 (13)	0.0442 (13)	0.0314 (12)	−0.0069 (10)	0.0120 (10)	0.0030 (10)
C24	0.0269 (12)	0.0476 (14)	0.0356 (13)	−0.0003 (10)	0.0108 (10)	−0.0067 (11)
C25	0.0327 (12)	0.0303 (12)	0.0441 (14)	0.0061 (10)	0.0096 (10)	−0.0025 (10)
C26	0.0289 (12)	0.0259 (11)	0.0377 (12)	0.0000 (9)	0.0110 (10)	0.0003 (9)

### Geometric parameters (Å, º)

**Table:**

S1—C2	1.746 (2)	C22—C23	1.391 (4)
S1—C5	1.718 (3)	C23—C24	1.383 (3)
S2—C2	1.655 (2)	C24—C25	1.380 (4)
N3—N4	1.372 (3)	C25—C26	1.393 (3)
N3—C1	1.478 (3)	C1—H1	0.9500
N3—C2	1.356 (3)	C5—H5	0.9500
N4—C5	1.287 (3)	C12—H12	0.9500
C1—C11	1.519 (3)	C13—H13	0.9500
C1—C21	1.523 (3)	C14—H14	0.9500
C11—C12	1.385 (3)	C15—H15	0.9500
C11—C16	1.384 (3)	C16—H16	0.9500
C12—C13	1.382 (4)	C22—H22	0.9500
C13—C14	1.381 (4)	C23—H23	0.9500
C14—C15	1.376 (4)	C24—H24	0.9500
C15—C16	1.389 (4)	C25—H25	0.9500
C21—C22	1.390 (3)	C26—H26	0.9500
C21—C26	1.388 (3)		
			
S1···N4	2.552 (2)	C23···H23^vi^	3.0200
S2···C23^i^	3.689 (3)	C23···H5^iv^	2.8100
S2···H1	2.7600	C24···H5^iv^	2.8400
S2···H23^i^	2.9200	C24···H15^viii^	3.0200
S2···H25^ii^	3.0400	C25···H5^iv^	2.9500
S2···H1^iii^	3.0200	C26···H5^iv^	3.0300
S2···H12^iii^	3.1900	H1···S2	2.7600
N3···S1	2.5023 (17)	H1···H12	2.4200
N4···C22	3.334 (3)	H1···H22	2.4700
N4···S1	2.552 (2)	H1···S2^iii^	3.0200
N4···C16	3.320 (3)	H5···N4^iv^	2.8600
N4···C5^iv^	3.345 (3)	H5···C21^iv^	3.0100
N4···C26	3.413 (3)	H5···C22^iv^	2.8900
N3···H16	2.5800	H5···C23^iv^	2.8100
N4···H16	2.7200	H5···C24^iv^	2.8400
N4···H5^iv^	2.8600	H5···C25^iv^	2.9500
N4···H13^v^	2.6000	H5···C26^iv^	3.0300
C5···N4^iv^	3.345 (3)	H12···H1	2.4200
C5···C24^iv^	3.542 (3)	H12···S2^iii^	3.1900
C12···C26	3.422 (3)	H13···N4^ix^	2.6000
C16···N4	3.320 (3)	H14···C23^x^	3.0900
C22···N4	3.334 (3)	H15···C24^xi^	3.0200
C23···C23^vi^	3.540 (3)	H16···N3	2.5800
C23···S2^vii^	3.689 (3)	H16···N4	2.7200
C24···C5^iv^	3.542 (3)	H22···H1	2.4700
C26···C12	3.422 (3)	H22···H23^vi^	2.5700
C26···N4	3.413 (3)	H23···S2^vii^	2.9200
C11···H26	2.5800	H23···C22^vi^	2.9300
C11···H24^i^	3.1000	H23···C23^vi^	3.0200
C12···H26	3.0500	H23···H22^vi^	2.5700
C15···H24^i^	3.0200	H24···C11^vii^	3.1000
C16···H26	3.0200	H24···C15^vii^	3.0200
C16···H24^i^	3.0000	H24···C16^vii^	3.0000
C21···H5^iv^	3.0100	H25···S2^x^	3.0400
C22···H23^vi^	2.9300	H26···C11	2.5800
C22···H5^iv^	2.8900	H26···C12	3.0500
C23···H14^ii^	3.0900	H26···C16	3.0200
			
C2—S1—C5	89.76 (10)	C21—C26—C25	120.50 (19)
N4—N3—C1	118.15 (16)	N3—C1—H1	107.00
N4—N3—C2	118.15 (16)	C11—C1—H1	107.00
C1—N3—C2	123.70 (17)	C21—C1—H1	107.00
N3—N4—C5	109.62 (19)	S1—C5—H5	122.00
N3—C1—C11	112.39 (16)	N4—C5—H5	122.00
N3—C1—C21	109.30 (16)	C11—C12—H12	120.00
C11—C1—C21	114.05 (16)	C13—C12—H12	120.00
S1—C2—S2	125.74 (12)	C12—C13—H13	120.00
S1—C2—N3	106.91 (14)	C14—C13—H13	120.00
S2—C2—N3	127.35 (16)	C13—C14—H14	120.00
S1—C5—N4	115.57 (17)	C15—C14—H14	120.00
C1—C11—C12	117.54 (18)	C14—C15—H15	120.00
C1—C11—C16	123.42 (19)	C16—C15—H15	120.00
C12—C11—C16	119.0 (2)	C11—C16—H16	120.00
C11—C12—C13	120.7 (2)	C15—C16—H16	120.00
C12—C13—C14	119.9 (2)	C21—C22—H22	120.00
C13—C14—C15	119.8 (2)	C23—C22—H22	120.00
C14—C15—C16	120.3 (3)	C22—C23—H23	120.00
C11—C16—C15	120.2 (2)	C24—C23—H23	120.00
C1—C21—C22	118.23 (18)	C23—C24—H24	120.00
C1—C21—C26	122.67 (17)	C25—C24—H24	120.00
C22—C21—C26	119.10 (19)	C24—C25—H25	120.00
C21—C22—C23	120.2 (2)	C26—C25—H25	120.00
C22—C23—C24	120.3 (2)	C21—C26—H26	120.00
C23—C24—C25	119.8 (3)	C25—C26—H26	120.00
C24—C25—C26	120.1 (2)		
			
C5—S1—C2—S2	−179.44 (16)	C21—C1—C11—C12	−76.1 (2)
C5—S1—C2—N3	0.18 (16)	C21—C1—C11—C16	101.9 (2)
C2—S1—C5—N4	−0.3 (2)	N3—C1—C21—C26	108.4 (2)
C1—N3—N4—C5	179.84 (19)	C1—C11—C12—C13	177.6 (2)
N4—N3—C1—C11	90.8 (2)	C12—C11—C16—C15	0.1 (4)
N4—N3—C1—C21	−36.9 (2)	C16—C11—C12—C13	−0.6 (4)
C2—N3—N4—C5	−0.1 (3)	C1—C11—C16—C15	−178.0 (2)
C2—N3—C1—C21	143.07 (18)	C11—C12—C13—C14	0.8 (4)
N4—N3—C2—S1	−0.1 (2)	C12—C13—C14—C15	−0.5 (4)
N4—N3—C2—S2	179.55 (16)	C13—C14—C15—C16	0.0 (4)
C1—N3—C2—S1	179.96 (14)	C14—C15—C16—C11	0.3 (4)
C1—N3—C2—S2	−0.4 (3)	C1—C21—C22—C23	179.23 (18)
C2—N3—C1—C11	−89.3 (2)	C26—C21—C22—C23	0.1 (3)
N3—N4—C5—S1	0.3 (3)	C1—C21—C26—C25	−179.32 (18)
N3—C1—C11—C12	158.78 (19)	C22—C21—C26—C25	−0.2 (3)
N3—C1—C11—C16	−23.2 (3)	C21—C22—C23—C24	0.1 (3)
C11—C1—C21—C22	162.48 (18)	C22—C23—C24—C25	−0.1 (3)
C11—C1—C21—C26	−18.4 (3)	C23—C24—C25—C26	0.0 (3)
N3—C1—C21—C22	−70.8 (2)	C24—C25—C26—C21	0.2 (3)

Symmetry codes: (i) *x*−1, *y*, *z*; (ii) −*x*+1/2, *y*+1/2, −*z*+3/2; (iii) −*x*, −*y*+1, −*z*+1; (iv) −*x*+1, −*y*+1, −*z*+2; (v) *x*+1/2, −*y*+1/2, *z*+1/2; (vi) −*x*+1, −*y*+1, −*z*+1; (vii) *x*+1, *y*, *z*; (viii) *x*+1/2, −*y*+1/2, *z*−1/2; (ix) *x*−1/2, −*y*+1/2, *z*−1/2; (x) −*x*+1/2, *y*−1/2, −*z*+3/2; (xi) *x*−1/2, −*y*+1/2, *z*+1/2.

### Hydrogen-bond geometry (Å, º)

Cg2 is the centroid of the C21–C26 phenyl ring.

**Table:**

*D*—H···*A*	*D*—H	H···*A*	*D*···*A*	*D*—H···*A*
C1—H1···S2	0.95	2.76	3.181 (2)	108
C16—H16···N3	0.95	2.58	2.897 (3)	100
C5—H5···*Cg*2^iv^	0.95	2.74	3.670 (3)	157
C13—H13···N4^ix^	0.95	2.60	3.495 (3)	157

Symmetry codes: (iv) −*x*+1, −*y*+1, −*z*+2; (ix) *x*−1/2, −*y*+1/2, *z*−1/2.
